# Prevalence, Awareness, Treatment, and Control of Hypertension in Romania: Results of the SEPHAR Study

**DOI:** 10.4061/2010/970694

**Published:** 2010-02-01

**Authors:** M. Dorobantu, R. O. Darabont, E. Badila, S. Ghiorghe

**Affiliations:** ^1^Department of Cardiology, Emergency Hospital Floreasca, 8 Calea Floreasca, 014461 Bucharest, Romania; ^2^Department of Cardiology, University Emergency Hospital, 050098 Bucharest, Romania

## Abstract

East European countries have reported high prevalence of Arterial Hypertension (AHT). In order to investigate the data for Romania, we firstly performed a national survey—the Study for the Evaluation of Prevalence of Hypertension and Cardiovascular Risk in Adult Population in Romania (SEPHAR). A representative population was selected using stratified proportional sampling, including 2017 adult subjects, ≥18 years old. The general prevalence of AHT was 44,92%, higher in men (50,17%) than in women (41,11%) (*P* < .0001) and predominant in rural areas (49,47%) in comparison to the urban ones (41,58%) (*P* < .02). AHT awareness attended 44,26%, rising with age, significantly lower in men (34,58%) than in women (52,8%) (*P* < .0006). We have found a 38,85% proportion of treated hypertensive persons, worse for men (30,11%) then for women (46,56%) (*P* < .003). The rate of AHT control was 19,88%, with no significant differences between gender. In conclusion, we estimated for Romania a high prevalence of AHT, a level of awareness and treatment lower than in many European countries and a rate of treatment control at the inferior limit of the European average. Males, characterized by a higher prevalence of AHT, were also less aware and less treated than women.

## 1. Introduction

The population impact of arterial hypertension (AHT) consists in its high prevalence and in the strength of its risk ratio with cardiovascular pathologic conditions. More than a quarter of the world adult population is already hypertensive and this number is projected to increase to 1,56 billion people by 2025. This estimation relies especially on a steep increase of AHT in the developing countries [1, 2]. 

A large number of clinical trials have shown that the cardiovascular risk could be considerably diminished by a tight reduction of the blood pressure (BP) values. Despite their encouraging data, the real rates of arterial hypertension awareness, and control are continuing to remain unsatisfactory in many areas of the world [3, 4]. 

Romania is an East European developing country, with a high mortality in cardiovascular disease, as was reconfirmed in the last European Cardiovascular Disease Statistics report [5] and it is supposed that uncontrolled AHT is an important contributor to this result. In order to find out the prevalence, the awareness, and the control of blood pressure in Romania, we have carried on a cross-sectional survey, named SEPHAR: Study for the Evaluation of Prevalence of Hypertension and cardiovascular risk in Adult population in Romania. This study is the first assessment of blood pressure and major correlated cardiovascular risk factors accomplished on a representative sample for the entire adult population in Romania.

## 2. Method

The design of the SEPHAR study was similar with that of the NATPOL PLUS study, conducted earlier in Poland [6, 7]. The data of our study were collected during the year 2005. 

The procedures applied in the SEPHAR study were in accordance with the Declaration of Helsinki of 1975, revised in 1983.

The selection of the subjects was based on the method of *stratified proportional sampling*. Romania's area was divided into ten regions, recommended by The National Commission of Statistics. The locations of interviewing were selected at random, on a basis of a computerized system elaborated and applied by a company specialized in research. The structure of the population aged ≥18, according to sex, in each location, was obtained from the Central Bureau of Statistics, having as a source document the census made in 2002. The addresses of interviewing were selected from the database of the General Direction of Computerized Evidence of the Population. Consequently, we were able to find out that in a specific location there is a person, male or female, aged of certain years. It is worth noticing that, using this procedure, we did not reach a person with a precise identity; we found out a subject with certain demographic characteristics, with respect to the Law no. 677/2001 on protection of persons regarding the processing of personal data and their free circulation in Romania. 

 On the whole, 2131 subjects agreed to participate in the study, to have the blood pressure measured, and to fill in the medical history questionnaires. Among these, 2017 have accepted also the collection of blood samples. 

Specially trained nurses obtained data for the respondents at the patients' home. During the visit, the nurse had to obtain the informed consent for the participation in the study, to complete the questionnaire, and to measure the arterial blood pressure, weight, height, and waist circumference. The BP was measured at each arm with fully automatic devices (oscillometric pressameters OMRON M5-I), adapted for the arm circumference. The highest value between the two arms was that of reference. If at the initial visit the BP was elevated and the subject was not known with arterial hypertension, two more visits for BP measurement were mandated to confirm the abnormal values, at one-to-three day interval. Three measurements of BP had to be done at each visit with at least three minutes pause between the consecutive readings, ignoring the first one and calculating the average between the last two of them. The arterial HT was defined as values ≥140 mmHg for the SBP or ≥90 mmHg for the DBP, according to the European Society of Hypertension Guidelines applicable in 2005 [8] and reconfirmed by the Guidelines issued in 2007 [9]. *Awareness* represented the subjects with known arterial hypertension. *New diagnosed cases of arterial hypertension *were those who have denied a previous diagnosis of hypertension and were found at the initial and at the confirmation visits with high BP. *The prevalence of arterial hypertension *resulted from the addition of known to the new diagnosed cases. *The general control of hypertension* was calculated from the ratio of subjects with blood pressure below 140/90 mmHg to the number of hypertensive persons on a whole (known and newly diagnosed). The *control of treated hypertension* was deduced from the ratio of subjects with blood pressure below 140/90 mmHg to the total number of treated hypertensive persons.

## 3. Statistical Analysis

The statistical evaluation was performed with a MEDCALC 9.2.1.0 software. Data about the prevalence, awareness, rate of treatment, and control of hypertension were obtained for the whole sample and by comparing specific categories of population splitted by gender, age groups, and area of residence with the *χ* ^2^ test.

On the analysis of the results, the statistical significance was tested with an interval of confidence of 95%, for which the maximum error was of ±2,18% for 2017 respondents.

## 4. Results

The age and gender structure, the repartition of subjects depending on the area of residence, and the mean values of blood pressure are represented in Table 1.

We have to mention that Bucharest, the capital, which was analyzed separately from the other nine regions of the country, was included in the urban area, conducting to a higher number of subjects in this category of residence.

The mean systolic BP was 137.63 ± 23.43 mmHg, with an important rise for every age group in each gender. The values have continuously increased from 119.99 ± 13.70 mmHg in the youngest age group to 152.76 ± 24.17 mmHg in the age group ≥65 years old. The mean diastolic BP was 83.13 ± 13.12 mmHg, higher with every age group until the age of 55–64 years, but starting to decline in the oldest age group (≥65 years). For both systolic and diastolic BP, the mean level was higher in men than in women for adult subjects, the gender difference diminishing by the sixth decade.

### 4.1. Prevalence of Hypertension

The total prevalence of the arterial hypertension was 44,92%, significantly higher in men (50,17%) than in women (41,11%) (*P* < .0001), but with the gender difference beginning to diminish in the age group of 55–64 years. The frequency of arterial hypertension was augmenting with age, reaching a rate above 70% after 65 years. A greater prevalence of arterial hypertension was observed in the rural area (49,47%) as compared to the urban area (41,58%), which attended statistical significance (*P* < .02) Table 2.

### 4.2. Awareness of Hypertension

The results regarding the hypertension awareness are resumed in Table 3.

The general rate of awareness was 44,26%, increasing with age, higher in women (52,8%) than in men (34,58%) (*P* < .0006), with the gender difference being noticed for every age group after 45 years old. No differences in AHT awareness by area of residence were noticed.

### 4.3. Treatment of Hypertension

Only 38,85% of the hypertensive subjects were receiving specific treatment. The rate of treated hypertension was again in favor of women (46,56%) in comparison with men (30,11%) (*P* < .003) Table 4.

### 4.4. Control of Hypertension

The number of the total hypertensive subjects who were on the target values of BP was 7,72%. The analysis of data regarding the treatment control has revealed a general rate of 19,88%, with no differences between gender or area of residence Table 4.

## 5. Discussion

SEPHAR was the first study addressed to the prevalence, awareness, and control of hypertension on a representative sample for the entire population of Romania. Even if the selection of the subjects was based on the method of stratified proportional sampling, the results must be cautiously interpreted due to the limited number of subjects included, in comparison with the adult inhabitants as a whole.

When referring the data to the literature, we have to notice the heterogeneity of different studies with regard to the sampling method (stratified sampling or selection during clinical visits), age of subjects enrolled, number of visits for the measurements of blood pressure, adaptation of cuff size for the arm circumference, or the period of time when the survey has been done. Therefore, a special attention will be focused on the comparison with the results of NATPOL PLUS, a study that has been realized with the same methodology in Poland. 

Our data are indicating a 44,92% prevalence of the AHT in Romania, higher than in Poland, where was estimated at 29% [6, 7]. In general, the rates of AHT are greater in Europe than in the United States (28%), Canada (27%), or Asian countries including China (20%) or Korea (22,9%) [4, 10–12]. The prevalence of AHT in Romania can be integrated between lower values in Europe like those in Greece (31,1), Sweden (38%), Italy (38%), United Kingdom (42%), Czech Republic (42%), or Portugal (42,1%) and higher values such as those from Spain (47%) or Germany (55%) [4, 13–15]. For each gender, AHT was rising in successive age groups. In our study AHT was significantly more prevalent in men. The ratio of AHT frequency between the two genders is varying from one study to another and in Poland was greater in women. There are not many studies analyzing the results in regard to the area of residence. In contrast to other observations and the assertion that AHT is more often encountered in the urban areas, we have found a bigger prevalence of high blood pressure in the rural areas. We are not able to comment, at this moment, if these results could be explained by some deleterious habits in lifestyle such as the excessive salt or alcohol consumption or by concomitant predominance of obesity in the rural zones.

Less than half of the hypertensive subjects were aware of their condition. In the polish study, the awareness was 67% [6, 7]. In Europe, the hypertension awareness is varying from 46% in Portugal [15] to 60% in Greece [13] or even 70% in Czech Republic [14]. In countries with national health programs effectuated for cardiovascular risk reduction, the level of AHT awareness has much improved, rising to 66% in United Kingdom [16] or 76% in United States [10] and attesting the success of a coordinated policy for high BP detection. 

The rate of treatment control in our sample (19,88%) was similar with Poland and comparable with the results of other studies [4, 14, 17]. In Europe, BP control is varying usually between 20 to 30%. The GOOD survey has found that in the Atlantic European Mainland more patients had uncontrolled BP (80%) compared with Northwest, Mediterranean, and Central Europe (70%) [18]. Recent data from BP-CARE study, conducted in Central and East European Countries (including Romania) with subjects selected from clinical visits to general practitioners or specialists, are showing that BP control was achieved in only 27,1% of treated hypertensive persons [19]. 

As a whole, in comparison with the results of the NATPOL PLUS study in Poland, the prevalence of AHT was higher, the awareness was worse, and the BP control was similar. These data should be interpreted, in the future, taking into account the differences between the genetic background, the lifestyle habitudes, and the health policies in each country.

## Figures and Tables

**Table 1:** The sample structure in the SEPHAR study and the mean values of the systolic and diastolic BP values.

	Total	Men	Women
Number of subjects	2017	847	1170

Age groups			
18–24	160	70	90
25–34	340	145	195
35–44	352	135	217
45–54	428	176	252
55–64	328	123	205
≥65	409	198	211

Area of residence			
Urban	1164	485	679
Rural	853	362	491

Mean values of systolic blood pressure (mmHg)			

Total	137.56 ± 23.9	142.66 ± 49.77	134.97 ± 24.18
18–24	119.71 ± 13.88	126.77 ± 11.87	114.22 ± 12.85
25–34	123.71 ± 14.24	129.73 ± 15.27	119.24 ± 11.58
35–44	128.35 ± 17.31	133.45 ± 16.03	125.17 ± 17.36
45–54	139.62 ± 22.31	142.43 ± 22.17	137.66 ± 22.24
55–64	148.82 ± 23.65	150.09 ± 23.38	148.06 ± 23.84
≥65	152.79 ± 25.58	152.96 ± 26.59	152.48 ± 24.68

Mean values of diastolic blood pressure (mmHg)			

Total	83.07 ± 13.58	86.78 ± 43.66	82.44 ± 13.71
18–24	72.35 ± 9.38	73.11 ± 9.06	71.76 ± 9.63
25–34	76.55 ± 9.94	78.68 ± 10.64	74.96 ± 9.09
35–44	81.11 ± 11.17	82.56 ± 11.91	80.21 ± 11.51
45–54	86.17 ± 12.97	87.68 ± 13.72	85.12 ± 12.33
55–64	89.1 ± 14.65	88.98 ± 13.32	89 ± 15.42
≥65	86.28 ± 13.97	86.12 ± 13.58	86.38 ± 14.35

**Table 2:** Prevalence of arterial hypertension in SEPHAR study.

	Total		Men		Women	
	n/N	%	n/N	%	n/N	%

General prevalence of hypertension						

	906/2017	44,92%	425/847	50,17%	481/1170	41,11%

Prevalence of hypertension splitted by age groups						

18–24	14/160	8.75%	9/70	12.85%	5/90	5.55%
25–34	51/340	15%	34/145	23.44%	17/195	8.71%
35–44	99/352	28.12%	51/135	37.77%	48/217	22.11%
45–54	220/428	51.4%	102/176	57.95%	118/252	46.82%
55–64	215/328	65.54%	77/123	62.6%	138/205	67.31%
≥65	307/409	75.06%	152/198	76.76%	155/211	73.45%

Prevalence of hypertension splitted by area of residence						

Urban	484/1164	41.58%	226/485	46.6%	258/679	38%
Rural	422/853	49.47%	199/362	54.97%	223/491	45.42%

**Table 3:** Arterial hypertension awareness in SEPHAR study.

	Total		Men		Women	
	n/N	%	n/N	%	n/N	%

Awareness of hypertension splitted by age groups						

Total	401/906	44.26%	147/425	34.58%	254/481	52.8%
18–24	0/14	0%	0/9	0%	0/5	0%
25–34	2/51	3.92%	0/34	0%	2/17	11.76%
35–44	27/99	27.27%	14/51	27.45%	13/48	27.08%
45–54	94/220	42.72%	31/102	30.39%	63/118	53.38%
55–64	109/215	50.69%	30/77	38.96%	79/138	57.24%
≥65	169/307	55.04%	72/152	47.36%	97/155	62.58%

Awareness of hypertension splitted by area of residence						

Urban	230/484	47.52%	84/226	37.16%	146/258	56.58%
Rural	171/422	40.52%	63/199	31.65%	108/223	48.43%

**Table 4:** Rate of arterial hypertension treatment and control in SEPHAR study.

	Total		Men		Women	
	n/N	%	n/N	%	n/N	%

Treated cases of hypertension splitted by age groups						

Total	352/906	38.85%	128/425	30.11%	224/481	46.56%
18–24	0/14	0%	0/9	0%	0/5	0%
25–34	2/51	3.92%	0/34	0%	2/17	11.76%
35–44	20/99	20.2%	9/51	17.64%	11/48	22.91%
45–54	85/220	38.63%	30/102	29.41%	55/118	46.61%
55–64	94/215	43.72%	24/77	31.16%	70/138	50.72%
≥65	151/307	49.18%	65/152	42.76%	86/155	55.48%

Treated cases of hypertension splitted by area of residence						

Urban	202/484	41.73%	73/226	32.3%	129/258	50%
Rural	150/422	35.54%	55/199	27.64%	95/223	42.6%

Hypertension control for treated patients						

Total	70/352	19.88%	27/128	21.09%	43/224	19.19%

Hypertension control for treated patients by area of residence						

Urban	49/202	24.25%	19/73	26.02%	30/129	23.25%
Rural	21/150	14%	8/55	14.54%	13/95	13.68%

General control of hypertension						

Total	70/906	7.72%	27/425	6.35%	43/481	8.93%

General control of hypertension by area of residence						

Urban	49/484	10.12%	19/226	8.4%	30/258	11.62%
Rural	21/422	4.97%	8/199	4.02%	13/223	5.82%
